# Baicalin Attenuates IL-17-Mediated Acetaminophen-Induced Liver Injury in a Mouse Model

**DOI:** 10.1371/journal.pone.0166856

**Published:** 2016-11-17

**Authors:** Chia-Chih Liao, Yuan-Ji Day, Hung-Chen Lee, Jiin-Tarng Liou, An-Hsun Chou, Fu-Chao Liu

**Affiliations:** 1 Department of Anesthesiology, Chang Gung Memorial Hospital, Taoyuan, Taiwan; 2 College of Medicine, Chang Gung University, Taoyuan, Taiwan; 3 Graduate Institute of Clinical Medical Sciences, Chang Gung University, Taoyuan, Taiwan; University of Navarra School of Medicine and Center for Applied Medical Research (CIMA), SPAIN

## Abstract

**Background:**

IL-17 has been shown to be involved in liver inflammatory disorders in both mice and humans. Baicalin (BA), a major compound extracted from traditional herb medicine (Scutellariae radix), has potent hepatoprotective properties. Previous study showed that BA inhibits IL-17-mediated lymphocyte adhesion and downregulates joint inflammation. The aim of this study is to investigate the role of IL-17 in the hepatoprotective effects of BA in an acetaminophen (APAP)-induced liver injury mouse model.

**Methods:**

Eight weeks male C57BL/6 (B6) mice were used for this study. Mice received intraperitoneal hepatotoxic injection of APAP (300 mg/kg) and after 30 min of injection, the mice were treated with BA at a concentration of 30 mg/kg. After 16 h of treatment, mice were killed. Blood samples and liver tissues were harvested for analysis of liver injury parameters.

**Results:**

APAP overdose significantly increased the serum alanine transferase (ALT) levels, hepatic activities of myeloperoxidase (MPO), expression of cytokines (TNF-α, IL-6, and IL-17), and malondialdehyde (MDA) activity when compared with the control animals. BA treatment after APAP administration significantly attenuated the elevation of these parameters in APAP-induced liver injury mice. Furthermore, BA treatment could also decrease hepatic IL-17-producing γδT cells recruitment, which was induced after APAP overdose.

**Conclusion:**

Our data suggested that baicalin treatment could effectively decrease APAP-induced liver injury in part through attenuation of hepatic IL-17 expression. These results indicate that baicalin is a potential hepatoprotective agent.

## Introduction

Drug-induced liver injury can cause severe hepatotoxicity and even acute liver failure. Acetaminophen (APAP) overdose is the leading cause of life-threatening acute hepatotoxicity in humans and animals [1, 2]. Acetaminophen (*N*-acetyl-*p*-aminophenol) is eliminated majorly by conjugation with glucuronide or sulfate in hepatocytes at therapeutic dosages. The remaining APAP is metabolized by the cytochrome P450 system and convert into a highly toxic intermediate, N-acetyl-p-benzoquinone imine (NAPQI). NAPQI is depleted by conjugation with glutathione (GSH) and the complex is excreted mainly into bile [3]. However, at toxic doses of APAP, excessive NAPQI depletes the cellular storage of GSH and binds to cellular proteins covalently, resulting in mitochondrial dysfunction and DNA damage, which leads to hepatocyte necrosis and cell death [4, 5].

Upon damage to parenchymal hepatocytes, innate immunity participates in the progression and amplification of APAP-induced liver injury. The initiation is characterized by the release of pro-inflammatory cytokines and chemokines by activated Kupffer cells (resident hepatic macrophages), leading to the signal propagation characterized by the facilitation of the adhesion and transmigration of neutrophils into the hepatic vasculature, which aggravates liver injury [6].

There is growing evidence that interleukin-17 (IL-17) plays a pivotal role in the recruitment of inflammatory cells to injury sites and is associated with a wide range of inflammatory disorders in both mice models and human studies [7, 8]. It has been shown to exacerbate inflammatory responses. T lymphocytes infiltrated into injured tissue rapidly and regulated neutrophil recruitment via their release of IL-17 during the course of inflammation [9, 10]. IL-17 can be produced from CD4+ αβ T cells (T helper 17, Th17), γδT cells, and NK cells. Recent studies demonstrated that γδT cells are a major and potent source of IL-17 and are thought to orchestrate innate and adaptive immune function [11]. IL-17-producing γδT cells can exacerbate autoimmune diseases [12]. In addition, neutrophil recruitment to the site of inflammation is mediated by γδT cells in a sepsis model [13]. Previous studies also showed that, in toxin-induced liver injury, there was a significant increase in IL-17 expression and a correlation between neutrophil infiltration and IL-17–secreting cells [14, 15].

Baicalin (5,6,7-trihydroxyflavone-7-beta-D-glucuronic acid, BA) is a major flavonoid compound isolated from Scutellariae radix, an important herb used in traditional medicine. Its safety has been clinically proven, and it possesses anti-allergic, antioxidant, and anti-inflammatory properties [16, 17]. Previous studies also reported that baicalin has hepatoprotective effects on liver injury models in mice [18, 19]. In addition, baicalin inhibits IL-17-mediated lymphocyte adhesion and downregulates joint inflammation [20]. However, the effect of baicalin administration in APAP-induced liver injury is not yet reported. Whether IL-17 plays any role in baicalin-related hepatoprotective function is unknown. Therefore, the aim of this study was to investigate the role of the IL-17 signaling pathway in hepatoprotective effects of baicalin in a mouse model of APAP-induced liver injury.

## Materials and Methods

### Animals

Eight weeks male C57BL/6 (B6) mice were used for this study. All strains of mice were maintained in B6 background in these studies. The mice were purchased from BioLASCO Taiwan Co., Ltd. (Taipei, Taiwan). All animal experiments were performed according to the guidelines of the *Animal Welfare Act* and the *Guide for Care and Use of Laboratory Animals* from the National Institutes of Health. All procedures and protocols were approved by the Institutional Animal Care and Use Committee of Chang Gung Memorial Hospital.

### Experimental model and drug treatment

All animals were housed in an environmentally controlled room, under pathogen-free conditions, with a 12-hour light and 12-hour dark cycle, and allowed free access to food and clean water during the experiments. Twenty-four male mice (24–27 g) were randomly divided into 4 groups (n = 6/group). APAP (Sigma Chemical Co., St. Louis, MO, USA) was dissolved in normal saline at a concentration of 20 mg/mL. The mice received an intraperitoneal hepatotoxic injection of APAP (300 mg/kg) and the control group received an equal volume of normal saline. After 30 minutes of injection, the mice were intraperitoneally injected with BA (Sigma) at a concentration of 30 mg/kg or an equal volume of phosphate-buffered saline (PBS). Then mice were sacrificed after 16 hours of APAP exposure. In another experiment for oxidative stress, mice were sacrificed 2, 6, 16 and 24 hours after the APAP exposures. Furthermore, for experimental studies into liver regenerative outcome, mice were sacrificed at 16, 24, 48, 72, and 96 hours after APAP administration. At each time point, all animals were killed by cervical dislocation under isoflurane anesthesia. Blood samples were drawn from the vena cava into syringes, and livers were harvested for further analysis.

### Measurement of APAP-induced hepatotoxicity

Blood samples were obtained at the end of the experiment (16 hours treatment) and immediately centrifuged at 12000 *g* for 5 minutes. Serum levels of alanine aminotransferase (ALT) were measured to determine hepatic injury by using a Vitros DT60 II Chemistry System (Ortho-Clinical Diagnostics; Johnson & Johnson, New York, NY). All the procedures and sample processing were according to the manufacturer’s manual.

### Measurement of liver myeloperoxidase (MPO) activity

Myeloperoxidase is released from the neutrophils into the phagosome and extracellular space. It is now recognized as an inflammatory indicator. Liver tissues of mice were homogenized with a Tekmar tissue grinder and centrifuged at 15000 *g* for 15 minutes at 4°C. The pellet was resuspended in 50 mM KPO_4_ buffer, pH 6.0, with 0.5% hexadecyltrimethylammonium bromide, incubated for 2 hours and sonicated by the sonicator (QSONICA Q700). The suspension was centrifuged at 15000 *g* for 15 minutes at 4°C. Then, the supernatant was transferred to phosphate buffer containing *o*-dianisidine hydrochloride (10 mg/mL), 0.3% hydrogen peroxide, and 50 mM KPO_4_, pH 6.0. The change in light absorbance was measured at 460 nm for a period of 5 minutes.

### Measurement of TNF-α, IL-6, and IL-17 levels

The liver tissues were homogenized and centrifuged at 12000 *g* for 10 minutes at 4°C. The supernatants were collected and analyzed for TNF-α, IL-6, and IL-17 expression using the eBiosciences ELISA Kit (San Diego, CA, USA) following the manufacturer’s instructions. Briefly, the 96 well plates were precoated with primary antibodies and incubated with 50 ug/100 uL sample for 2 hours. After washing several times, biotinylated secondary antibodies were added for 1 hour. Then, after incubation with HRP substrate for 30 minutes, the absorbance was measured at 450 nm using TECAN infinite 200.

### Flow cytometric analysis

Immunophenotyping detection was done by direct immunofluorescence using multicolor flow cytometry staining of isolated total leukocytes. Livers were harvested, passed through a 70-μm cell strainer, and leukocyte fractions were isolated via Percoll density gradient. Leukocytes were isolated from mice for analysis of the γδT cell population change upon APAP and BA treatment. Cells were washed and resuspended at 2 × 10^5^ cells/mL in PBS. Cellular surface was stained with PE–conjugated anti-mouse TCRγδ (eBioscience) for 1 hour. The fluorescence intensity was measured with a cytomics FC500 flow cytometer (Beckman Coulter, Fullerton, CA). A minimum of 8000 FACS events were recoeded for each sample. An excitation wavelength of 488 nm and an emission wavelength of 575±15 nm were used for detection of PE-stained cells. Analysis was performed with FlowJo software (Tree Star, Inc.).

### Lipid peroxidation assay

We measured the generation of malondialdehyde (MDA) as the indicator of lipid peroxidation using a Bioxytech MDA-586 Kit (OxisResearch, Portland, OR, USA). Briefly, the supernatants of liver homogenate were mixed with probucol, *N*-methyl-2-phenylindole, and hydrochloric acid to produce a stable dye. After centrifugation, the absorbance of the clear supernatant was measured using a spectrometry at 586 nm. The concentration of MDA was plotted against a standard curve and expressed in nmole per milligram of wet tissue.

### Superoxide dismutase (SOD) assay

SOD activity was determined according to the manufacturer’s instructions (Cayman Chemical Company, MI, USA). This assay utilizes a tetrazolium salt for the detection of superoxide radicals and converts tetrazolium salt to formazan. SOD decreases the concentration of superoxide radicals and thereby reduces the rate of formazan formation. Therefore, relative SOD activity, expressed as U/mg protein, is measured by inhibition of the rate of formazan dye formation.

### Histology

Livers were harvested, fixed in 4% paraformaldehyde in PBS pH 7.4, and embedded in paraffin. Tissue sections (5 μm) were subjected to standard hematoxylin and eosin (H&E) staining for light microscopy. To measure the necrotic area, tissue sections were viewed under a light microscope (Zeiss Axioskop). Photographs were taken and adjustment with a SPOT RT camera (software version 3.3; Diagnostic Instruments). The percent of necrosis area was evaluated from five microscopic views with necrosis compared with the entire field.

### Immunohistochemistry on liver tissue

Livers were harvested, fixed in a commercial fixed buffer (fineFix), and embedded in paraffin. The paraffin blocks were sliced into 4-μm serial sections and de-paraffinized. Liver samples were blocked with blocking buffer for 30 minutes, and then incubated with a specific primary antibody against IL-17 overnight. After washing for 5 minutes twice, samples were incubated with biotinylated secondary antibodies for 1 hour. The peroxidase reaction was performed following the manufacturer’s protocol (Millipore IHC select kit). The reaction times for all sections from experimental animals were identical. Additional liver sections were stained for detection of proliferating cell nuclear antigen (PCNA) (Cell Signaling Technology, MA, USA). Positively stained hepatocytes were quantified by randomly selecting five high power fields.

### Western blotting assay

Liver samples were obtained at each time points and the homogenate was made with ice-cold lysis buffer. The cell lysates were separated by 10% sodium dodecylsulfate polyacrylamide gel (SDS-PAGE) and transferred to polyvinylidene fluoride (PVDF) membrane. After blocking with 5% fat-free milk solution, the membrane was incubated overnight at 4°C with anti-PCNA primary antibody (Cell Signaling Technology, MA, USA). After washing with horseradish peroxidase-conjugated secondary antibody, proteins were detected using the enhanced chemiluminescence system.

### Statistical analysis

All data were expressed as mean ± SEM. Statistical calculations were performed with GraphPad Prism 5.0 Software (GraphPad Software Inc., San Diego, USA). The results were analyzed using unpaired t tests or one-way analysis of variance (ANOVA) with post-hoc Tukey multiple comparison tests. Differences were considered statistically significant between groups if *p* < 0.05.

## Results

### Effects of treatment with baicalin on serum ALT levels

**Fig 1:**
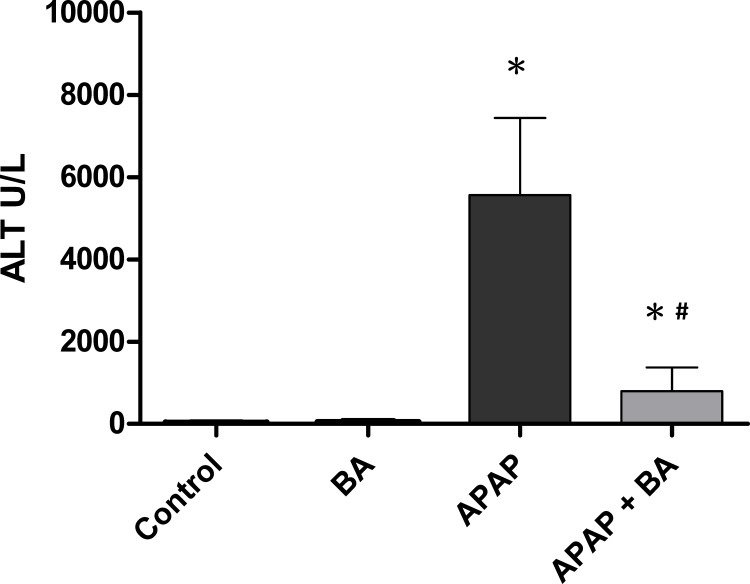
Effects of treatment with baicalin against APAP-induced hepatotoxicity: Mice received an intraperitoneal hepatotoxic injection of APAP (300 mg/kg) alone, or treatment with BA (30 mg/kg) 30 minutes after APAP injection. All mice were killed 16 hours after administration for analysis of serum ALT. Each value is mean ± SEM of six mice per group. *p < 0.05 *vs*. control; ^#^ *p <* 0.05 *vs*. APAP alone.

Serum levels of ALT are shown in Fig 1. There was no significant difference in the serum ALT enzyme activity between control and BA-only treated groups. Serum ALT levels were significantly elevated in the group receiving a single dose APAP (300 mg/kg) when compared with the control group (5566.7 ± 764.2 vs. 26.8 ± 5.5 U/L, *p* < 0.05), which confirmed the hepatotoxicity of APAP overdose. After 30 minutes of APAP administration, treatment with BA (30 mg/kg) markedly decreased serum ALT levels. Serum ALT level was significantly attenuated in BA treatment group compared with the APAP-only group (798.7 ± 233.9 vs. 5566.7 ± 764.2 U/L, *p <* 0.05).

### Effects of treatment with baicalin on hepatic MPO activity

**Fig 2:**
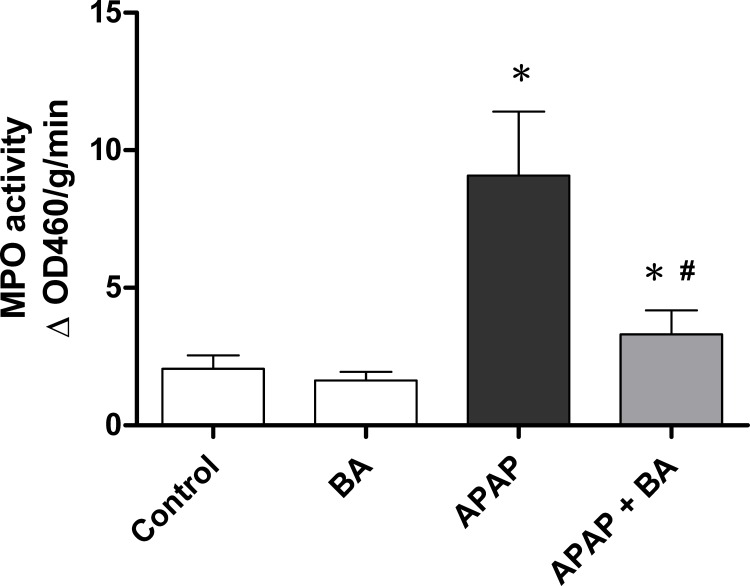
Effects of treatment with baicalin on hepatic MPO activity in APAP-induced liver injury: Mice received an intraperitoneal hepatotoxic injection of APAP (300 mg/kg) alone, or treatment with BA (30 mg/kg) 30 minutes after APAP injection. All mice were killed 16 hours after administration for analysis of liver MPO activity. Each value is mean ± SEM of six mice per group. *p < 0.05 *vs*. control; ^#^ *p <* 0.05 *vs*. APAP alone.

Liver MPO activity is a marker of inflammatory response and neutrophil accumulation. Hepatic expression of MPO is shown in Fig 2. There was no significant difference between control and BA-only treated groups. It was significantly elevated in the group receiving a single dose of APAP (300 mg/kg) when compared with the control animals (9.0849 ± 0.9455 vs. 2.0546 ± 0.1999 #x00B1;OD460/g/min, *p* < 0.05). After 30 minutes of APAP administration, treatment with BA (30 mg/kg) attenuated hepatic MPO levels. Hepatic MPO activity was significantly decreased in BA treatment group compared with the APAP-only group (3.3111 ± 0.3534 vs. 9.0849 ± 0.9455 ±OD460/g/min, *p <* 0.05).

### Effects of treatment with baicalin on hepatic TNF-α and IL-6 levels

**Fig 3:**
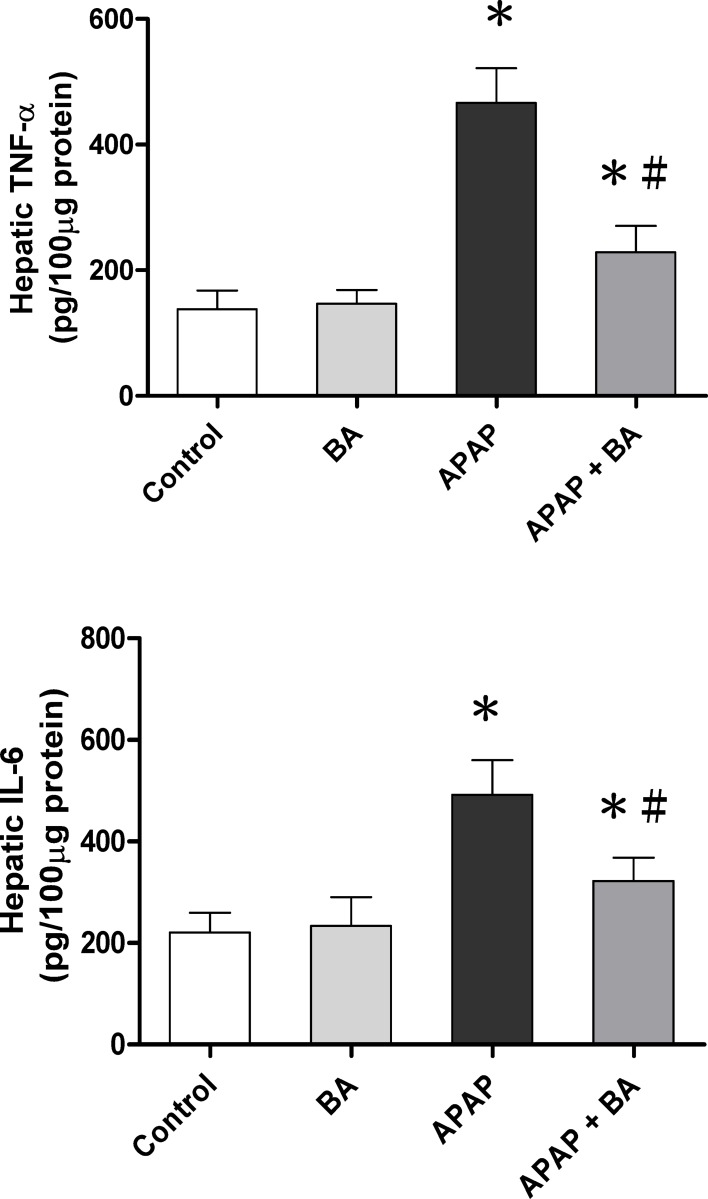
Effects of treatment with baicalin on hepatic TNF-α and IL-6 levels in APAP-induced liver injury: Mice received an intraperitoneal hepatotoxic injection of APAP (300 mg/kg) alone, or treatment with BA (30 mg/kg) 30 minutes after APAP injection. All mice were killed 16 hours after administration for analysis of liver TNF-α and IL-6 levels. Each value is mean ± SEM of six mice per group. *p < 0.05 *vs*. control; ^#^ *p <* 0.05 v*s*. APAP alone.

As shown in Fig 3, we investigated the levels of pro-inflammatory cytokines including TNF-α and IL-6, after APAP-induced liver injury. The results demonstrated no significant difference in hepatic TNF-α and IL-6 levels between the control and BA-only treated groups. In the APAP group, the concentrations of TNF-α and IL-6 were significantly increased after single dose administration of APAP (300 mg/kg) for 16 hours compared with the control group (466.2 ± 22.8 vs. 138.3 ± 12.0 pg/100μg protein, *p <* 0.05; 491.6 ± 27.9 vs. 221.2 ± 15.6 pg/100μg protein, *p <* 0.05, respectively). However, treatment with BA (30 mg/kg) after 30 minutes of APAP overdose significantly decreased elevated hepatic TNF-α and IL-6 levels compared with the APAP alone treated group (228.9 ± 17.1 vs. 466.2 ± 22.8 pg/100 μg protein, *p <* 0.05; 322.2 ± 18.8 vs. 491.6 ± 27.9 pg/100μg protein, *p <* 0.05, respectively).

### Effects of treatment with baicalin on hepatic IL-17 levels

**Fig 4:**
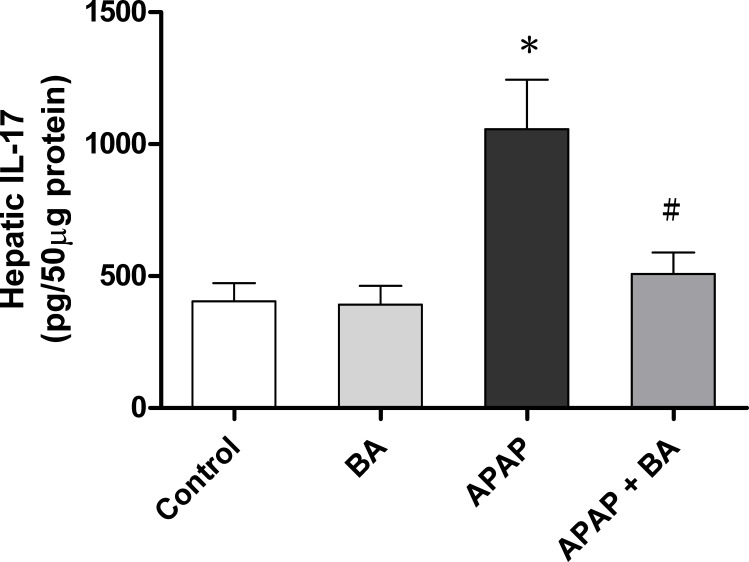
Effects of treatment with baicalin on hepatic IL-17 levels in APAP-induced liver injury: Mice received an intraperitoneal hepatotoxic injection of APAP (300 mg/kg) alone, or treatment with BA (30 mg/kg) 30 minutes after APAP injection. All mice were killed 16 hours after administration for analysis of liver IL-17 levels. Each value is mean ± SEM of six mice per group. *p < 0.05 *vs*. control; ^#^ *p <* 0.05 v*s*. APAP alone.

As shown in Fig 4, we investigated the immunoregulatory activity of IL-17. The results demonstrated no significant difference in hepatic IL-17 expression between the control and BA-only treated groups. In the APAP group, the levels of IL-17 were significantly increased after single dose administration of APAP (300 mg/kg) for 16 hours compared with the control group (1056.4 ± 188.6 vs. 404.5 ± 68.1 pg/50 μg protein, *p <* 0.05). However, treatment with BA (30 mg/kg) after 30 minutes of APAP overdose markedly decreased elevated hepatic IL-17 level compared with the APAP alone treated group (507.6 ± 81.9 vs. 1056.4 ± 188.6 pg/50 μg protein, *p <* 0.05). The IL-17 level in the APAP plus BA treated group had nearly returned to baseline values compared with the control group.

### Effects of treatment with baicalin on immunostaining for IL-17

**Fig 5:**
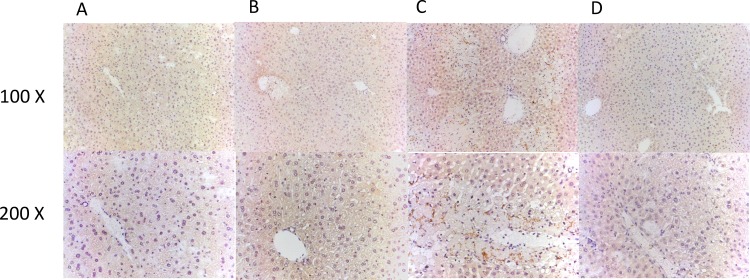
**Effects of treatment with baicalin on immunohistological changes of IL-17 expression in APAP-induced liver injury:** Mice received (A) control (normal saline), (B) BA (30 mg/kg) alone, (C) APAP (300 mg/kg) alone, or (D) BA (30 mg/kg) after 30 minutes of APAP injection. All mice were killed 16 hours after administration for analysis by immunohistochemistry. Liver tissues were immunostained with anti–IL-17 Ab (brown). Representative images were chosen from each group (100× and 200× magnification is shown).

Immunohistochemistry staining of liver tissue from the control mice displayed normal hepatic architecture as shown in Fig 5A. Immunohistochemical staining showed increased IL-17 staining around the area of necrosis in the liver parenchyma in APAP-only treated group compared to the APAP plus BA treated group (Fig 5C and 5D). The results suggested that BA treatment after APAP injection significantly decreased IL-17 expression around the inflammatory area of hepatotoxicity in the liver. Immunofluorescence staining showed similar trends in the liver parenchyma (S1 Fig.).

### Effects of treatment with baicalin on γδT cell recruitment

**Fig 6:**
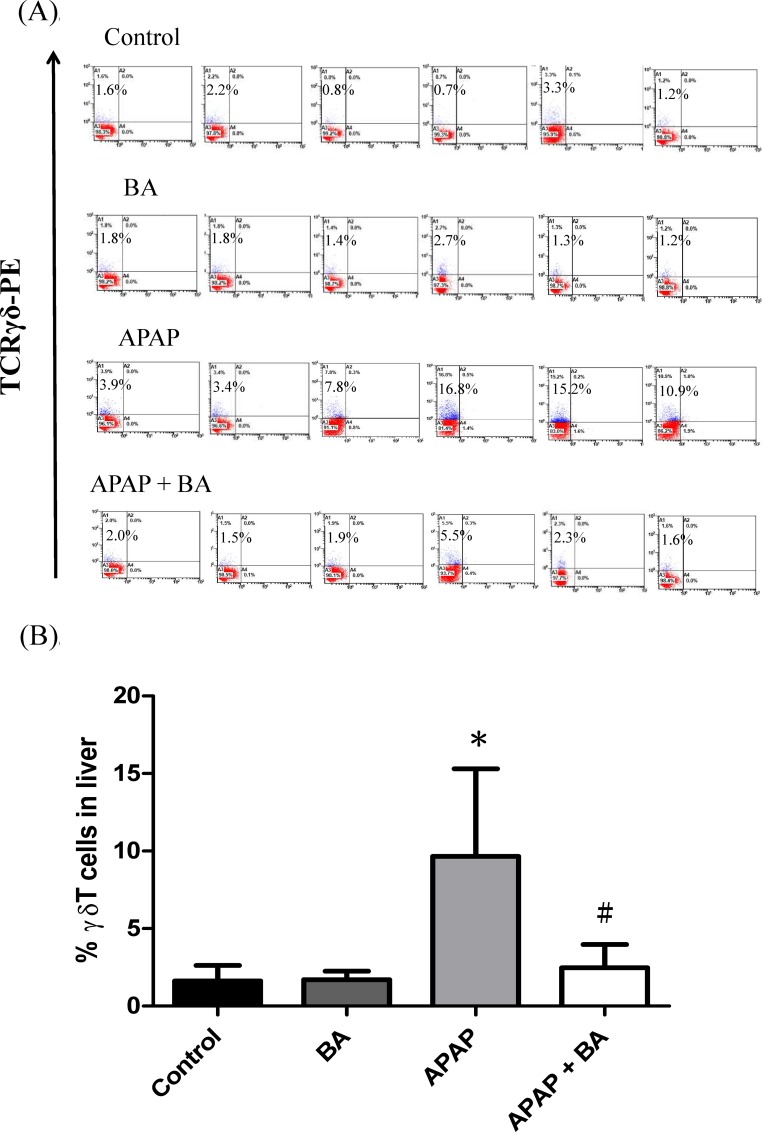
**Effects of treatment with baicalin on flow cytometry analysis of γδT cells expression in APAP-induced liver injury:** Representative plots demonstrate TCRγδ-positive T cells (A) and quantification of flow cytometry data (B). Mice received an intraperitoneal hepatotoxic injection of APAP (300 mg/kg) alone, or treatment with BA (30 mg/kg) 30 minutes after APAP injection. All mice were killed 16 hours after administration for analysis of liver γδT cells accumulation. Each value is mean ± SEM of six mice per group. *p < 0.05 *vs*. control; ^#^ *p <* 0.05 v*s*. APAP alone.

IL-17 is produced by specific γδT cells, which induce pro-inflammatory protein expression and recruit neutrophils. Flow cytometric features and quantitative analysis of γδT cells are shown in Fig 6A and 6B. There were no significant differences in hepatic γδT cell accumulations between the control and BA-only treated groups. The results of flow cytometry analysis demonstrated that the infiltration of γδT cell was significantly increased in the APAP group compared with the control group after single dose of APAP administration (9.7 ± 2.3 vs. 1.6 ± 0.4, *p <* 0.05). However, BA treatment (30 mg/kg) after 30 minutes of APAP administration significantly declined hepatic γδT cell recruitment. Hepatic γδT cell expression was significantly decreased in APAP plus BA treated group compared with the APAP-only group (2.5 ± 0.6 vs. 9.7 ± 2.3, *p <* 0.05).

### Effects of treatment with baicalin on MDA and SOD activities

**Fig 7:**
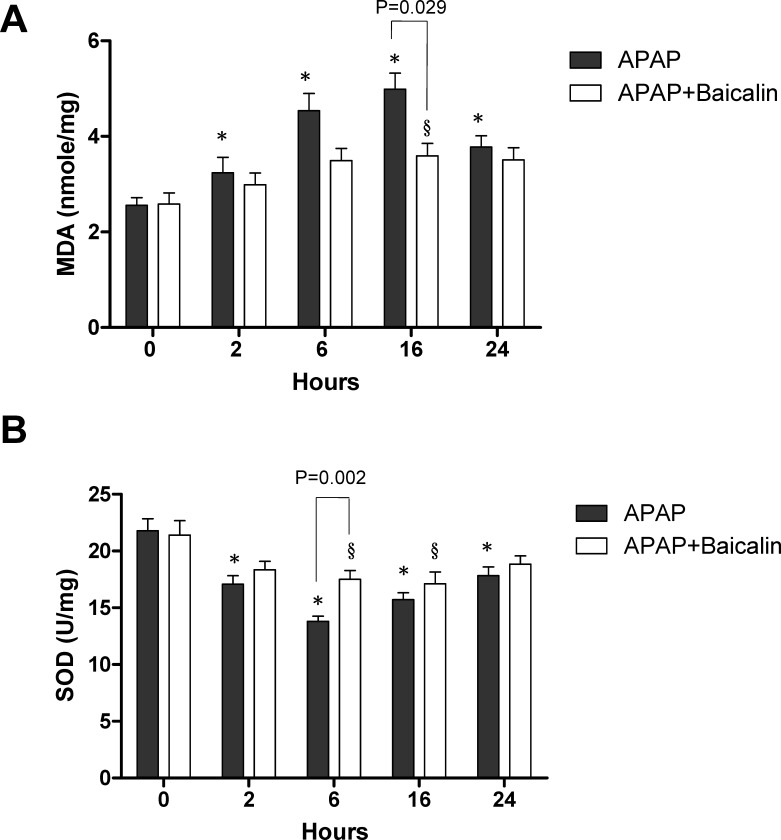
Effects of treatment with baicalin on MDA and SOD activities in APAP-induced liver injury: Mice received an intraperitoneal hepatotoxic injection of APAP (300 mg/kg) alone, or treatment with BA (30 mg/kg) 30 minutes after APAP injection. Time course of (A) hepatic MDA and (B) SOD activities was measured after administration with APAP. Each value is mean ± SEM (n = 5–6 animals per group at each time point). *p < 0.05 *vs*. APAP control group; ^§^*p* <0.05 v*s*. APAP plus BA control group.

APAP overdose causes the production of oxidative stress. We measured MDA levels as lipid peroxidation product (Fig 7A). The concentration of MDA was significantly increased at each time points in APAP-only treated group and at 16 h in APAP plus BA treated group when compared with their control group (0 hour). In addition, there were significant differences between the 2 groups at 16 hours after APAP exposure (*p* = 0.029). Next, we determined the antioxidant activity of SOD (Fig 7B). APAP administration significantly diminished SOD activities compared with the control group. However, at 6 hours after APAP exposure in BA treatment group, there was significantly alleviated APAP-induced SOD depletion (*p* = 0.002).

### Effects of treatment with baicalin on histology

**Fig 8:**
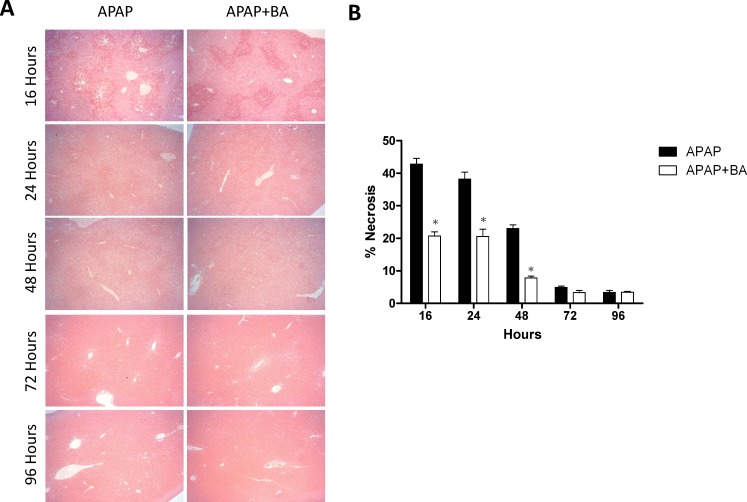
Effects of treatment with baicalin on histological changes in liver injury and regeneration: Mice received an intraperitoneal hepatotoxic injection of APAP (300 mg/kg) alone, or treatment with BA (30 mg/kg) 30 minutes after APAP injection. All samples were obtained at 16, 24, 48, 72, and 96 h after APAP exposures. (A) Representative images of H&E staining were chosen from each group (50× magnification is shown). (B) Bar graph showed quantitative analysis of the percentage of necrotic area. Each value is mean ± SEM of five mice per group. *p < 0.05 *vs*.the APAP group.

We compared liver injury of two groups over the time course of 16 to 96 hours by histopathological analysis (Fig 8A and 8B). Liver injury was characterized by centrilobular necrosis and fatty infiltration as evidenced in H&E staining. After APAP administration, liver injury markedly increased and peaked at 16 hours. There were significantly increased necrotic areas at 16, 24, and 48 hours in APAP-only group compared with the APAP plus BA treated group. The overall time course of hepatic injury showed similar trend in both groups and gradual recovery of injury after 48 hours.

### Effects of treatment with baicalin in liver regeneration

**Fig 9:**
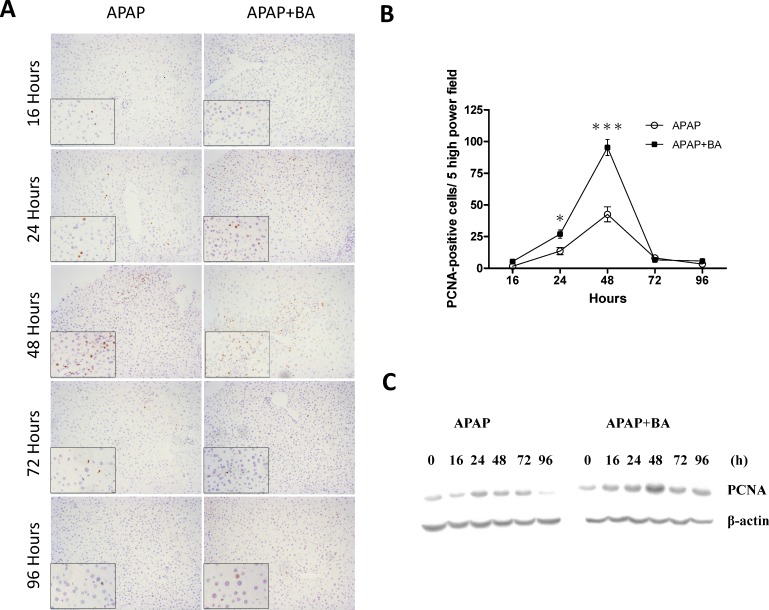
Effects of treatment with baicalin in liver regeneration after APAP-induced liver injury: Mice received an intraperitoneal hepatotoxic injection of APAP (300 mg/kg) alone, or treatment with BA (30 mg/kg) 30 minutes after APAP injection. All samples were obtained at 16, 24, 48, 72, and 96 h after APAP exposures. (A) Liver tissues were immunostained with anti–PCNA Ab (brown). Representative images were chosen from each group (100× and 200× magnification is shown). (B) Line graph showed quantitative analysis of total PCNA-positive cells. (C) Hepatic PCNA protein expression. Each value is mean ± SEM of five mice per group. *p < 0.05, * * *p < 0.001 *vs*. the APAP group.

PCNA is a cell regeneration marker that assists in DNA replication. We compared liver regeneration of two groups over the time course of 16 to 96 hours and detected PCNA expression by immunohistochemistry staining of liver tissue as shown in Fig 9A. Quantitative analysis of all PCNA-positive cells (Fig 9B) showed that there were obviously increased proliferation responses from 24 hours after APAP exposure in both groups. The total numbers of PCNA-positive cells remain progressively elevated and peaked at 48 hours, and gradually decreased at 72 and 96 hours. Interestingly, there were significantly more cell proliferations observed at 24 and 48 hours in BA-treated group compared with the APAP-only group. Hepatic PCNA protein expressions by western blot analysis are shown in Fig 9C. The results further corroborated the data from immunohistochemical staining.

## Discussion

Acetaminophen is a widely used analgesic and antipyretic drug in clinical practice. However, its overdose can induce severe liver injury and even liver failure. In the present study, the protective effects of baicalin, a flavonoid compound purified from Scutellariae radix, on APAP-induced liver injury in a mouse model were studied. After APAP administration for 16 hours to induce liver injury, increases in serum ALT concentrations, hepatic MPO activity, pro-inflammatory cytokines, and oxidative stress parameters were observed. Treatment with baicalin after 30 minutes of APAP administration significantly displayed attenuated increase in these hepatic parameters. In addition, this protective effect may correlate with the attenuation of hepatic IL-17 expression and IL-17-producing γδT cells recruitment. These findings collectively imply that IL-17 contributes to the hepatoprotective effects of baicalin.

Our results demonstrated that baicalin alleviated the process of inflammation in APAP-induced liver injury. APAP hepatotoxicity is characterized by aseptic inflammation, and innate immune mechanisms play a critical role in amplifying inflammatory responses. Excessive electrophilic NAPQI are reported to cause direct hepatocyte death, and Kupffer cells were induced in concert with subsequent activation of inflammatory cytokines and infiltration of leukocytes into the liver [21]. TNF-α and IL-6 play an important role in the regulation of acute inflammatory response [22]. Recent studies reported that neutrophils mediated direct injury and amplified hepatocytes death during APAP-induced acute liver failure [23]. The induction of TNF-α and IL-6, and increased neutrophil accumulation have also been well described in some drug-induced or ischemia-reperfusion liver injury models [24, 25]. Therefore, cytokines expression and neutrophil infiltration are central mediators of inflammation. On the other hand, previous evidence showed that baicalin inhibited inflammatory responses via down-regulation of cytokines in an animal model with myocardial ischemic injury [26]. Another report also showed that baicalin suppressed the production of cytokines and leukocyte migration in *in vitro* and *in vivo* studies [27]. In our study, early treatment with baicalin after APAP overdose could effectively decrease hepatic cytokine levels accompanied by MPO activity. It indicated that baicalin treatment after APAP-induced liver injury attenuated inflammatory mediators and neutrophil infiltration.

NAPQI depletes the cellular storage of GSH and the formation of free radicals results in APAP toxicity [4, 5]. Previous studies have demonstrated that baicalin exerts hepatoprotective effects via inhibiting oxidative stress in alcohol-induced hepatotocixity [28]. Another study also showed that baicalin could attenuate cerebral ischemia/reperfusion injury by anti-oxidative pathway [29]. Our results confirmed that APAP overdose could increase MDA level and reduce SOD activity, indicating accumulation of reactive oxygen species and production of lipid peroxidation. These APAP-induced changes in MDA level and SOD activity was reduced in mice treated with baicalin, suggesting that baicalin directly contributed to decrease oxidative stress.

Our data showed that the expression of IL-17 was markedly increased after APAP overdose. Recently, there is growing evidence that IL-17 plays an important role in enhancing immune responses in human liver diseases and in autoimmunity [7, 8]. In human alcoholic liver disease, patients had higher plasma IL-17 levels compared with healthy subjects [14]. In halothane-induced liver injury, the increases in plasma IL-17 levels and neutrophil infiltration were also observed. Inhibition of the IL-17 pathway might offer protection from the hepatotoxic effect [15]. Furthermore, it has been reported that IL-17 induced the expression of inflammation-associated genes including chemokines and C-reactive protein in hepatocytes [30]. A previous study also showed that IL-17 stimulated expression of pro-inflammatory cytokines/chemokines produced by liver nonparenchymal cells including monocytes, Kupffer cells, biliary epithelial cells, and stellate cells, which contributed to liver inflammation [31].

In addition, IL-17 exerts effects on neutrophils. During ischemia-reperfusion injury, T lymphocytes regulated hepatic neutrophil recruitment through the production of IL-17 [32]. IL-17 can regulate inflammation through coordinated expression of many inflammatory cytokines/chemokines in neutrophil activation and infiltration. It induced the release of neutrophil-mobilizing cytokine, including IL-6 and IL-8, to site of infection [33]. It was also found to mediate neutrophil chemotaxis by increasing the expression of the chemokine macrophage inflammatory protein 2 (MIP-2) in a model of peritoneal inflammation [34]. These data suggested an important role of IL-17 via its effects on neutrophils in immune and inflammatory responses. A recent study has demonstrated that baicalin reduced joint inflammatory injury caused by IL-17 and blocked IL-17-mediated expression of adhesion molecules in murine experimental arthritis [20]. Our results showed that baicalin decreased the expression of hepatic IL-17 levels and neutrophil recruitment. It implies that baicalin attenuated inflammatory response through reduced production of IL-17 in APAP-induced liver injury.

IL-17 has shown to be involved in the induction and mediation of inflammatory responses. It can be produced by many types of cells including Th17, γδT, and NK cells. Th17 lymphocytes represent a newly identified T helper cell lineage and have a pro-inflammatory role in neutrophilic inflammation by IL-17 production. However, further recent studies demonstrated that γδT cells are a major and potent source of IL-17 and play an important role link between innate and adaptive immune functions [11]. In some previous studies, γδT cells have been shown to be more dominant and produced higher amounts of IL-17 than Th17 cells [35, 36]. Therefore, in our reports, we examined γδT cells as major IL-17-producing cells. During the course of inflammatory responses, γδT cells have innate cell-like characters that allow earlier activation and more rapid production of IL-17 than Th17 [37]. Even γδT cells can secrete IL-17and IL-21, which promote further IL-17 production by Th17 cells in an animal model of central nervous system autoimmunity [38]. Another study also showed that antibody depletion of γδT cells led to decreased IL-17 production and neutrophil infiltration after bacterial infection [39]. In human viral hepatitis, patients display higher numbers of γδT cells in the liver when compared to healthy subjects [40]. The functional role of γδT cells seemed to be associated with the progression of organ injury [41, 42]. Our results showed that the accumulation of γδT cells is increased following APAP-induced liver injury than in the control group. Treatment with baicalin decreased the infiltration of γδT cells in liver tissues. These data suggested that baicalin administration attenuated the expansion of these IL-17-producing T cells.

Baicalin has been used in traditional herb medicine for many years due to its anti-inflammatory and antioxidant properties. Previous studies showed that baicalin down-regulates the function of macrophages and inhibits the production of inflammatory mediators released by macrophages in a rat model [43]. The anti-inflammation effect of baicalin on ulcerative colitis was associated with the inhibition of TLR4/NF-κB signaling pathway [44]. A recent study found that the TLR4-dependent pathway in macrophages mediates the generation of IL-17-producing γδT cells and subsequent inflammation in an APAP-induced liver inflammation model [45]. In our study, we demonstrated that baicalin treatment effectively reduced liver injury after APAP overdose by attenuation of hepatic γδT cells and IL-17 expression. Collectively, this implied that baicalin elicits protective effects in APAP-induced liver injury through the cascading of macrophages and γδT cells. In addition, we detected significant differences in PCNA expression in the liver tissues and confirmed the effects of baicalin on liver cell regeneration. Further studies are needed to investigate the effects of baicalin on this regenerative pathway of liver.

## Conclusions

Our study showed that baicalin treatment could suppress accumulation of γδT cells and exert hepatoprotective effects via attenuation of IL-17-mediated inflammation. It could effectively decrease APAP-induced liver injury in part by inhibition of hepatic IL-17 expression and its effectors, including inflammatory markers and neutrophil infiltration. Taken together, these findings indicated that baicalin may potentially develop as a hepatoprotective drug and can be considered as a promising therapeutic agent for APAP-induced liver injury in humans. Further studies regarding its clinical applications are required to be explored.

## Supporting Information

S1 Fig Effects of treatment with baicalin on immunofluorescence evidence of IL-17 expression in APAP-induced liver injury. The effects of baicalin on IL-17 expression in liver tissues are demonstrated by immunofluorescence analysis. Mice received (A) control (normal saline), (B) BA (30 mg/kg) alone, (C) APAP (300 mg/kg) alone, or (D) BA (30 mg/kg) after 30 minutes of APAP injection, and were killed 16 hours after treatment for immunofluorescence staining. (DAPI: blue; IL-17: red; Macrophage: green) Representative images were chosen from each group (400× magnification).
(TIF)
