# Supplementary figures and images for: Baicalin Attenuates IL-17-Mediated Acetaminophen-Induced Liver Injury in a Mouse Model
Source: PLoS One. 2016 Nov 17;11(11):e0166856. doi: 10.1371/journal.pone.0166856 (PMC5113979; doi:10.1371/journal.pone.0166856)

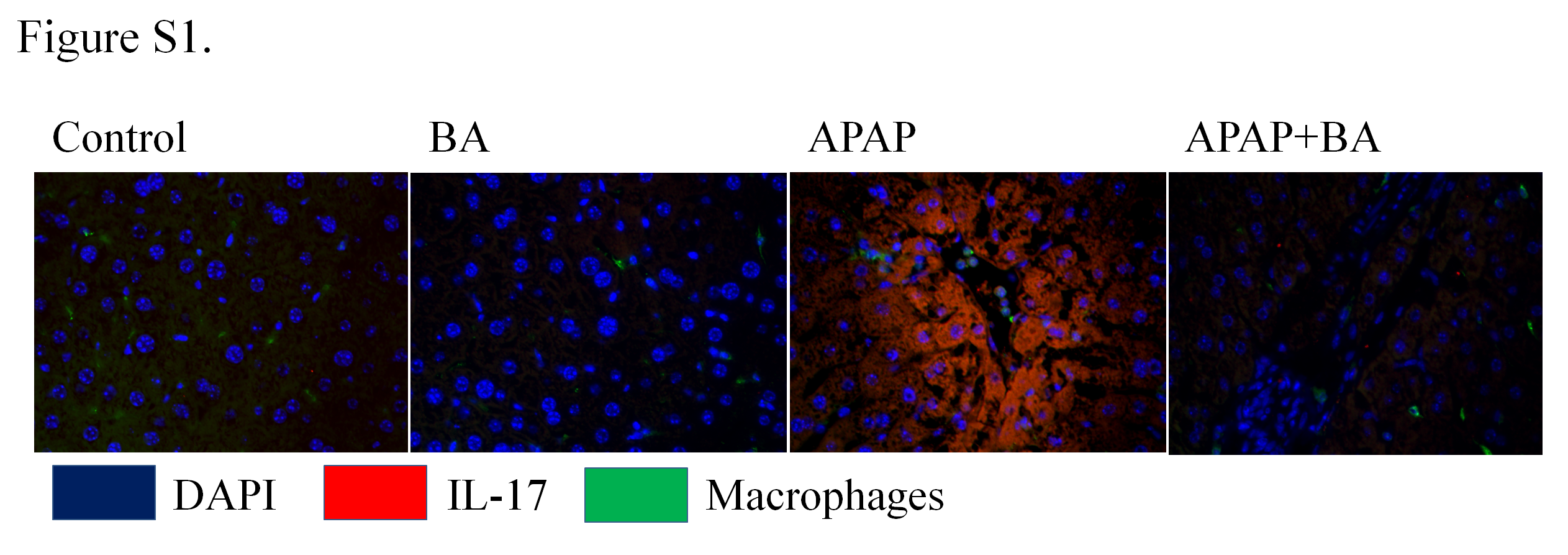

Supplement: S1 Fig — The effects of baicalin on IL-17 expression in liver tissues are demonstrated by immunofluorescence analysis. Mice received (A) control (normal saline), (B) BA (30 mg/kg) alone, (C) APAP (300 mg/kg) alone, or (D) BA (30 mg/kg) after 30 minutes of APAP injection, and were killed 16 hours after treatment for immunofluorescence staining. (DAPI: blue; IL-17: red; Macrophage: green) Representative images were chosen from each group (400× magnification). (TIF) [file pone.0166856.s001.tif]
